# Homöopathie – eine Therapieoption für die Praxis? Bewertung unter dem Blickwinkel der evidenzbasierten Medizin

**DOI:** 10.1007/s00106-021-01061-w

**Published:** 2021-05-19

**Authors:** Christian W. Lübbers, Udo Endruscheit

**Affiliations:** 1 HNO Weilheim, Pöltnerstraße 22, 82362 Weilheim, Deutschland; 2 Krayer Straße 227, 45307 Essen, Deutschland

**Keywords:** Qualitätssicherung im Gesundheitswesen, Berufsethik, Systematische Übersichtsarbeit, Placeboeffekt, Medizinethik, Health care quality assurance, Professional ethics, Systematic review, Placebo effect, Medical ethics

## Abstract

Viele Veröffentlichungen bezeichnen die Homöopathie als „umstritten“. Angesichts umfangreicher Forschungsergebnisse zur Homöopathie besteht jedoch längst weitestgehend wissenschaftlicher Konsens dahin, dass es keinen belastbaren Beleg für eine spezifische medizinische Wirksamkeit gibt. Die Gesamtevidenz spricht klar gegen Effekte, die über die von Placebo- und anderen Kontexteffekten hinausgehen. Umso mehr muss es als Phänomen erscheinen, dass die Homöopathie nach wie vor Gegenstand medizinisch-therapeutischer Praxis ist. Dies mag eine wesentliche Ursache darin haben, dass sich die homöopathische Szene der medizinischen Forschung und des Evidenzbegriffs auf eine Weise bemächtigt, die geeignet ist, den Anschein aufrechtzuerhalten, es gäbe noch einen wissenschaftlich relevanten Diskurs zu bestreiten. Dass dies nicht der Fall ist und deshalb die Homöopathie als therapeutische Option, auch nach den Grundsätzen zeitgemäßer Medizinethik, obsolet ist, will der nachstehende Beitrag begründen.

## Abstract (en)

Many publications declare homeopathy to be “controversial.” However, based on the findings of extensive research on homeopathy, there has long been a broad scientific consensus that there is no reliable evidence of specific medical effectiveness. Overall, the evidence clearly denies effects beyond those of placebo and context. All the more must it be seen as a phenomenon that homeopathy is still the subject of medical and therapeutic practice. This may lie largely in the fact that the homeopathic scene appropriates medical research and the concept of evidence in a way that is suitable to maintain the appearance that there is still a scientifically relevant discourse to dispute. The following article aims to justify that this is not the case, and that homeopathy is, therefore, obsolete as a therapeutic option, even according to the principles of contemporary medical ethics.

## Lernziele

Nach Lektüre dieses Beitrags …

- ist Ihnen der Begriff der Gesamtevidenz einer Intervention im Sinne der evidenzbasierten Medizin (EbM) vertraut,
- können Sie die Entwicklung der Gesamtevidenz aus dem Body of Evidence einer Intervention beschreiben,
- können Sie die Bedeutung einzelner Studien für die Beurteilung von Evidenz für eine Intervention beschreiben,
- kennen Sie die Gesamtevidenz für die Methode Homöopathie und die Arbeiten der höchsten Evidenzklasse, auf die sie sich stützt,
- kennen Sie den Zusammenhang zwischen Evidenz und Leitlinien als korrespondierende Elemente des Systems der EbM,
- sind Sie sich über die expliziten wie die implizierten Probleme der Anwendung von Homöopathie in der ärztlichen Praxis im Klaren.

## Hintergrund

Die Homöopathie spielt nach wie vor eine Rolle in der medizinischen Praxis. Die Hals-Nasen-Ohren-Heilkunde ist dabei keine Ausnahme. Neben der Frage nach dem realen therapeutischen Wert der Homöopathie und der Legitimität der Option, sie als Placebo oder Überbrückungsmedikation einzusetzen, stellt sich auch die nach einer möglichen Eignung von Homöopathika als Antibiotika-Alternative. Nach 30 Jahren empirischer Forschung unter den Prämissen der evidenzbasierten Medizin (EbM) [1] ist es an der Zeit, die Rolle der Homöopathie generell und in diesem praxisbezogenen Kontext kritisch und definitiv zu bewerten.

Das System der EbM zielt darauf ab, dass unter ausdrücklichem Verzicht auf Vorannahmen, Bedingungen und Plausibilitäten jedem Mittel und jeder Methode eine rein pragmatische, ideologiefreie Möglichkeit geboten wird, ihre **klinische Relevanz**

Klinische Relevanz

für die Behandlung von kranken Menschen zu belegen. Es zählt ausschließlich das Ergebnis (Outcome) des realen Nutzens für den Patienten. Damit ist die EbM – unabhängig von Plausibilitätsdiskussionen, Methodenstreitigkeiten und auch individuellen Erfahrungstatbeständen – der Königsweg, um die seit über 200 Jahren geführten Diskussionen über die Homöopathie auf den Kern, den **realen Nutzen**

Realer Nutzen

für die Patienten, zurückzuführen und zu einer definitiven Antwort zu gelangen. Teils weltanschaulich besetzte Berufungen auf einen „Pluralismus in der Medizin“ oder einem „Besten aus 2 Welten“, wie sie noch bis in die 2000er-Jahre hinein auch von Ärztefunktionären vertreten wurden, sind durch den rein ergebnisorientierten Ansatz der EbM obsolet geworden. Letztere bringt einen einheitlichen utilitaristischen Medizinbegriff^1^ zur Geltung: den einer nach wissenschaftlichen Maßstäben belegten **spezifischen Wirksamkeit**

Spezifische Wirksamkeit

über Kontexteffekte hinaus mit realem Nutzen für die PatientInnen.

Die Nullhypothese der Homöopathie ist die Existenz einer spezifischen arzneilichen Wirkung – die Überlegenheit über Placebo – unter den Prämissen der elementaren homöopathischen Grundannahmen^2^ (Ähnlichkeitsprinzip, Wirkungszunahme durch „Potenzieren“). Unabhängig von Auseinandersetzungen über diese Grundannahmen selbst bietet die EbM der Homöopathie eine „zweite Chance“^3^: nämlich die, ihre Hypothesen durch das wissenschaftlich fundierte Experiment – die Empirie – zu belegen. Erst wenn dieser Beleg belastbar erbracht würde, wäre es von Interesse, einen unbekannten Wirkungsmechanismus zu diskutieren.^4^

## Evidenz

### Merke

- Evidenz ist das, was sich aus systematisch wissenschaftlich bewerteten Quellen *insgesamt* für eine Methode oder ein Mittel ergibt.
- Was an der voraussetzungslosen Empirie der evidenzbasierten Methode scheitert, kann im fachlichen Kontext nicht mehr als „umstritten“ etikettiert werden.

### Body of Evidence

Etliche Studien und andere Materialien werden für und gegen eine Evidenz der Homöopathie bei verschiedensten Indikationen ins Feld geführt. Gemeinsam bilden sie den Body of Evidence. Die Gesamtheit der hier erfassten Erkenntnismaterialien als solche liefert aber noch keine belastbare Erkenntnis zur Evidenz. Ziel der EbM ist es, ausgehend vom Body of Evidence zu einer **Gesamtevidenz**

Gesamtevidenz

für eine medizinische Intervention zu kommen. Der Weg von einzelnen Ergebnissen zu belastbaren Erkenntnissen und letztlich bis hin zu einer Gesamtevidenz ist jedoch lang.

### Evidenzhierarchie

Als ersten Maßstab für die Validität des jeweiligen Erkenntnismaterials kennt die EbM eine Evidenzhierarchie. Dabei geht es um die Einordnung von Materialien nach ihrer **erwartbaren Validität**

Erwartbare Validität

auf der Grundlage ihrer jeweiligen Methodik und noch nicht um systematische konkrete Bewertungen. Die Hierarchien geben aber einen ersten Aufschluss darüber, welche Materialien vorrangig für eine Evidenzbegründung in Betracht kommen. Maßgeblich sind dabei stets diejenigen höherer Evidenzstufen. Das Heranziehen einer Quelle ohne Berücksichtigung ihrer Position in der Hierarchie ist irrelevant.

#### Merke

- Der einzelnen Quelle kommt nur eine sehr bedingte Bedeutung zu.
- Sie ist im günstigsten Falle ein *Hinweis* auf eine *möglicherweise vorhandene* Evidenz und bedarf auch hierzu genauer Prüfung ihrer Validität.
- Dies entspricht dem Ansatz der EbM, dem Kliniker für den Behandlungsfall „die gegenwärtig beste externe, wissenschaftliche Evidenz“ für die „Integration individueller klinischer Expertise mit der bestverfügbaren externen Evidenz aus systematischer Forschung“ verfügbar zu machen.

Evidenz ist kein Baukasten, aus dem nach Belieben Versatzstücke entnommen und passend kombiniert werden können. Die Evidenzbasierte Medizin versteht sich als ein fortlaufender Prozess insofern, als sie die *jeweils beste verfügbare externe Evidenz* für die medizinische Praxis verfügbar machen will. Aus dem Anspruch, die jeweils beste externe Evidenz zur Verfügung zu stellen, folgt ein breiter Evidenzbegriff, aber keine Beliebigkeit.

Viele in der Praxis verwendete Evidenzhierarchien beziehen sich auf das 5‑stufige, innerhalb der Stufen weiter differenzierte System des OCEBM Levels of Evidence 2 des Oxford Centre for Evidence Based Medicine [2]. Leicht modifiziert ist es Teil der Cochrane-Systematik zur EbM. Die Verfahrensrichtlinien des Gemeinsamen Bundesausschusses verwenden eine frühere Fassung mit etwas anderen Schwerpunkten [3].

#### OCEBM Levels of Evidence 2 des Oxford Centre for Evidence Based Medicine

- Evidenzstufe 1: systematische Übersichtsarbeit von randomisierten kontrollierten Studien,
- Evidenzstufe 2: randomisierte kontrollierte Studie oder Beobachtungsstudie mit dramatischem Effekt,
- Evidenzstufe 3: nichtrandomisierte kontrollierte Kohortenstudie,
- Evidenzstufe 4: Fallserien, Fall-Kontroll-Studien oder historisch kontrollierte Studien,
- Evidenzstufe 5: pathophysiologisch-mechanistische Argumente.

### Critical Appraisal

Die Gesamtheit der wissenschaftlichen Veröffentlichungen zu einem Thema, der Body of Evidence, ist praktisch immer mehr oder weniger heterogen, weshalb aus einzelnen Erkenntnismaterialien keine validen Schlüsse auf das Vorliegen von Evidenz gezogen werden dürfen, sondern allenfalls auf Hinweise (Indizien) hierfür geschlussfolgert werden kann. Die Belastbarkeit einer einzelnen Arbeit hängt von ihrer **internen Validität**

Interne Validität

ab, also davon, ob tatsächliche oder methodische Unzulänglichkeiten in Planung, Durchführung oder Auswertung das Ergebnis verzerren.

Wenn ausreichend geeignete Erkenntnismaterialien vorliegen, wird zur Gewinnung einer Gesamtevidenz für eine therapeutische Intervention dieser Body of Evidence qualitativ bewertet und gewichtet: Critical Appraisal. Dies geschieht mittels systematischer Reviews und Metaanalysen.^5^ In der Regel werden dafür möglichst alle verfügbaren Arbeiten der höchsten Evidenzstufe(n) herangezogen. Das Ziel solcher systematischen Arbeiten besteht in der qualitativ **gewichteten Darstellung**

Gewichtete Darstellung

des Body of Evidence: der Gewinnung einer Gesamtevidenz.

### Risk of Bias

Ein wesentlicher Maßstab für die Bewertung der Studien ist dabei der Risk of Bias, also das **Fehlerrisiko**

Fehlerrisiko,

das sich aus einer Beurteilung der einzelnen Komponenten von Planung, Design, Durchführung und Auswertung – der internen Validität – jeweils ergibt.

Die Analyse des Risk of Bias ist nicht beliebig, sondern systematisiert. Die Cochrane Collaboration stellt dafür innerhalb des **GRADE-Systems**

GRADE-System

(Grading of Recommendations Assessment, Development and Evaluation) Bewertungskriterien zur Verfügung [4], die eine Einordnung in 3 Qualitätsgruppen („high“, „unclear“ und „low risk of bias“) ermöglichen [5]. GRADE ist eine Methodik, um die Qualität der Evidenz für systematische Übersichtsarbeiten und für medizinische Empfehlungen (Leitlinien) zu systematisieren; sie wird international und auch von der Weltgesundheitsorganisation (WHO) angewandt.

Es liegt auf der Hand, dass in diesem Kontext einzelne – auch neu hinzutretende – Studien nur eine noch weiter eingeschränkte Bedeutung haben können. So muss ihre Belegkraft im Sinne von Carl Sagans Postulat „außergewöhnlich“ sein, um bereits auf hohem Evidenzniveau gesicherte Erkenntnis infrage stellen zu können.

#### Merke

- Bei genauer Betrachtung zeigen Homöopathiestudien nahezu regelhaft ihre mangelnde interne Validität.^6^
- Weiter wird ihre Belegkraft limitiert durch den α‑Fehler, falsch-positive Ergebnisse, die bei jeder Art von Untersuchung auftreten.
- Zudem fehlt ihnen durchweg die unabhängige Replikation, die einzige Methode, den rein statistischen Charakter einzelner Ergebnisse in Richtung realer Belastbarkeit zu festigen [6].
- So zeigen die systematischen Untersuchungen der Studienlage zur Homöopathie auch immer wieder, dass zunächst postulierte Effekte bei einer Beschränkung auf die methodisch besten Studien verschwinden.^7^

## Die systematischen Übersichtsarbeiten zur Homöopathie

Der Body of Evidence zur Homöopathie ist umfangreich. Er umfasst neben einer 3‑stelligen Zahl von **experimentellen Kontrollstudien**

Experimentelle Kontrollstudien

eine Vielzahl von Verlaufs- und **Beobachtungsstudien**

Beobachtungsstudien

sowie weitere Materialien. Auch systematische Reviews liegen in vergleichsweise hoher Zahl vor.

### Merke

- Seit 1991 sind insgesamt 11 größere indikationsübergreifende systematische Übersichtsarbeiten zum Body of Evidence der Homöopathie veröffentlicht worden.
- Keiner dieser 11 Reviews kam zu dem Ergebnis, es läge eine belastbare Evidenz für die Wirksamkeit einer homöopathischen Behandlung bei irgendeiner Indikation vor [7, 8, 9, 10, 11, 12].

Dieses einheitliche Ergebnis sei besonders betont, denn es ist völlig unabhängig von Studienauswahl, Methodik und Autorschaft der Übersichtsarbeiten. Einige stammen von Homöopathen, andere nicht. Ihre Schlussfolgerungen mögen mehr oder weniger klar formuliert sein, die Quintessenz ist jedoch immer gleich: belastbare Evidenz pro Homöopathie wurde nicht gefunden.

Es kann zudem keine Rede davon sein, es gebe „nicht genug Forschung“ zur Homöopathie. Es sollten doch 11 indikationsübergreifende **systematische Reviews**

Systematische Reviews

mit einheitlichen Schlussfolgerungen, die ihrerseits international von wissenschaftlichen und staatlichen Institutionen auf einer weiteren Metaebene analysiert und bestätigt wurden^8^, ausreichen, um die wissenschaftliche Bewertung der Homöopathie als abgeschlossen anzusehen, solange nicht wirklich „außergewöhnliche“ Gegenbelege auftauchen. Wobei Letzteres angesichts der bisherigen Empirie und der **unplausiblen Grundannahmen**

Unplausible Grundannahmen

der Homöopathie mit hinreichender Sicherheit nicht zu erwarten ist.

Insofern ist es ein besonderes Phänomen, dass unter diesen im Ergebnis letztlich nicht unterschiedlichen Untersuchungen zur Gesamtevidenz einerseits solche sind, die Vertreter der Homöopathie gleichwohl als positive Belege ins Feld führen, und andererseits auch solche, die von ihnen strikt zurückgewiesen und teils massiv attackiert werden. Dies soll nachstehend an 2 Beispielen aufgezeigt werden.

Das wohl häufigste Beispiel für eine Arbeit, die sinnwidrig, nämlich **selektiv zitierend**

Selektive Zitierung,

als Beleg pro Homöopathie angeführt wird, ist der Review von Mathie (2014) [13]: „Arzneien, die als Homöopathika individuell verordnet wurden, haben vielleicht einen kleinen spezifischen Effekt. (…) Die generell niedrige und unklare Qualität der Nachweise gebietet aber, diese Ergebnisse nur vorsichtig zu interpretieren.“ So Mathie in seinem eigenen Fazit.

Nahezu regelhaft lässt sich beobachten, dass aus dem vorstehenden Fazit nur der erste Satz zitiert und der zweite, der das ohnehin schwache Ergebnis nochmals stark relativiert, so gut wie nie angeführt wird.

Mathie betont zwar ausdrücklich, dass diese Arbeit den international geltenden Bewertungsregeln für klinische Studien voll Rechnung trage. Dies trifft jedoch nicht uneingeschränkt zu, die Studienauswahl und -bewertung von Mathie in dieser Arbeit ist zu Recht vielfach kritisiert worden.

Zu dem ohnehin nur knapp signifikanten Ergebnis eines „kleinen spezifischen Effektes“ kommt Mathie nur, indem er 2 von anderen Autoren als „low risk of bias“, also sehr zuverlässig bewertete, für die Homöopathie negative Arbeiten nicht berücksichtigt. Andererseits bezieht er 3 weniger valide Studien – darunter 2 Pilotstudien – in die Gesamtbewertung mit ein. Entsprechend neu bewertet zeigt auch diese so oft als Beleg pro Homöopathie angeführte Arbeit, dass die ohnehin geringen positiven Effekte vollständig verschwinden.

Obwohl im Endergebnis von gleicher Aussage wie Mathie (2014) und methodisch deutlich valider, ist andererseits die Veröffentlichung des **australischen National Health and Medical Research Council**

Australisches National Health and Medical Research Council

(NHMRC; 2015) [14] der wohl von homöopathischer Seite meistkritisierte der 11 Reviews. „Es gibt keine zuverlässigen Nachweise dafür, dass die Homöopathie bei der Behandlung von Gesundheitsproblemen bei irgendeiner Indikation wirkungsvoll wäre.“ So lautet das Fazit dieser wohl umfassendsten **systematischen Untersuchung**

Systematische Untersuchung

der Studienlage zur Homöopathie.

Das australische NHMRC (die Forschungseinrichtung des Gesundheitsministeriums) fand in 225 Studien über mehr als 80 Indikationen hinweg keine belastbaren Belege für eine spezifische Wirkung der Homöopathie.

Es ist kaum vorstellbar, mit mehr Akribie, mehr Transparenz und mehr Sorgfalt bei der Auswertung und Dokumentation vorzugehen als das NHMRC.

Nachdem sich erste Detailkritiken seitens der homöopathischen Szene als haltlos herausgestellt hatten [15], folgte die weltweit verbreitete Behauptung, es habe eine zulasten der Homöopathie „unterdrückte“ erste Version des Reports gegeben („First Draft“). Im August 2019 veröffentlichte die australische Regierung dann den angeblichen „First Draft“, wobei sich herausstellte, dass es sich lediglich um einen methodisch unhaltbaren Entwurf eines mit Auswertungsarbeiten beauftragt gewesenen Dienstleisters handelte. Die Gründe für das Verwerfen dieses nie von irgendjemand autorisierten Entwurfspapiers hatte das NHMRC in umfangreichen Anmerkungen zum „Draft“ und in einem Begleitschreiben der Direktorin des NHMRC erläutert [16]. Damit fand eine gegen eine hochrangige Dienststelle der australischen Bundesregierung in Szene gesetzte regelrechte **Verschwörungstheorie**

Verschwörungstheorie

nach und nach ihr Ende.

Eine neue Perspektive eröffnete dann eine Übersichtsarbeit aus neuerer Zeit, nämlich Antonelli/Donelli (2018) [17]: „Wenn die Wirksamkeit der Homöopathie mit einem Placebo vergleichbar ist und eine Behandlung mit Placebo bei manchen Beschwerden wirksam sein kann, dann kann man die Homöopathie insgesamt als Placebotherapie ansehen. Die Interpretation der Homöopathie als Placebotherapie definiert Grenzen und Möglichkeiten dieser Lehre.“ So die Autoren.

Wenn zu einem Mittel oder einer Methode über längere Zeit – wenn überhaupt – immer wieder nur geringe Signifikanzen und minimale klinische Effekte zutage treten, ist die Vermutung berechtigt, dass diese Effekte in der Realität gar nicht existieren. Das Auftreten solcher Signifikanzen und Effekte im Sinne **falsch-positiver Ergebnisse**

Falsch-positive Ergebnisse

(α-Fehler) in klinischen Forschungen ist wissenschaftliches Allgemeingut. Dass zudem die Häufigkeit des α‑Fehlers bei methodischen Unzulänglichkeiten und abhängig vom Grad der Plausibilität der Nullhypothese über den üblicherweise erwartbaren Wert hinaus ansteigt, wurde vielfach dargelegt [18]. 9Zumal bei fehlender Replizierung solcher Ergebnisse sind sie deshalb kein Anlass, stets und nachhaltig die Gesamtevidenz zu ignorieren. Daher ist der Schluss von Antonelli/Donelli auf den **Placebocharakter der Homöopathie**

Placebocharakter der Homöopathie

nur konsequent.

### Schlussfolgerung

Nichts deutet darauf hin, dass es derzeit real belastbare Nachweise einer über **Kontexteffekte**

Kontexteffekte

/Placeboeffekte hinausgehenden Wirksamkeit der Homöopathie bei der Behandlung von kranken Menschen gibt. Als ausgeschlossen kann gelten, dass die vielfach behauptete starke Leistungsfähigkeit der Homöopathie nachgewiesen wäre. Aussagen, die die Homöopathie als eine zur wissenschaftlichen Medizin gleichwertige, wenn nicht gar als eine überlegene Therapieform deklarieren, beruhen nicht auf wissenschaftlich gesicherten Belegen. Sie dürfen mit der in der empirischen Wissenschaft größtmöglichen erreichbaren Wahrscheinlichkeit als widerlegt angesehen werden.

#### Merke

Es gibt keine validen wissenschaftlichen Belege für eine über Kontexteffekte/Placebo hinausgehende Wirksamkeit von Homöopathika.

Dies ist die konsequente Schlussfolgerung aus den in zusammenfassenden Untersuchungen bewerteten empirischen Ergebnissen klinischer Forschung zur Homöopathie, ohne dass dafür die **fehlende Ausgangsplausibilität**

Fehlende Ausgangsplausibilität

(Scientabilität) bzw. die Unvereinbarkeit homöopathischer Prämissen mit naturgesetzlichen Gegebenheiten überhaupt diskutiert werden müsste. Die Evidenzbetrachtung nach den Kriterien der EbM, die mit ihrer Konzentration auf den **patientenrelevanten Outcome**

Patientenrelevanter Outcome

das fairste existierende medizinische Beurteilungssystem darstellt, ermöglicht ein abschließendes Urteil im Rahmen des epistemologisch Möglichen.^10^

## Homöopathische Forschung

Es stellt sich mit einiger Berechtigung die Frage, zu welchem Zweck eigentlich homöopathische Forschung nach wie vor betrieben wird. Zu Konsequenzen für die homöopathische Praxis führt sie ersichtlich nicht – weder für die Methode als solche noch für die therapeutische Praxis. Das widerstrebt dem prinzipiellen Forschungsansatz, dass eine Nullhypothese verworfen wird, wenn diese sich eben nicht bestätigen ließe.

Am Beispiel der **Münchner Kopfschmerzstudie**

Münchner Kopfschmerzstudie

(1997) [23], die keinerlei Überlegenheit der individuell verordneten Homöopathika über Placebogaben zeigte, lässt sich dies deutlich belegen. Kopfschmerzen sind unverändert eine der Hauptindikationen für Homöopathie in der Praxis und Gegenstand der Bewerbung homöopathischer Monopräparate und Komplexmittel. Wo soll also der Erkenntnisgewinn liegen? Die Fortführung homöopathischer Forschung und ihre Publikation – i. d. R. mit wenig aussagekräftigen einzelnen Arbeiten – wird jedenfalls die Fehlwahrnehmung weiter befördern, Homöopathie sei eine relevante medizinische Therapieform mit wissenschaftlichem Charakter, insbesondere dann, wenn dies entsprechend über die Medien transportiert wird [24].

Wenn es um den wissenschaftlichen Erkenntnisgewinn geht, kann nur mit Prof. Edzard Ernst konstatiert werden: „The debate about homeopathy is over.“ [25].

## Homöopathie in der Praxis

Es kann nicht ohne Auswirkungen auf die medizinische Praxis bleiben, wenn die Homöopathie ihren patientenrelevanten Nutzen für **keine Indikation**

Keine Indikation

belastbar belegen kann.

### Studien im HNO-Bereich

Unabhängig von ihrer Einordnung in die **negative Gesamtevidenz**

Negative Gesamtevidenz

werden nachfolgend beispielhaft einige Studien zu Krankheitsbildern der Hals-Nasen-Ohren-Heilkunde exemplarisch betrachtet. Es handelt sich um Arbeiten, die in der derzeit gültigen S2k-Leitlinie „Rhinosinusitis“ [26] erwähnt werden. Auch wenn sie dort nicht explizit als Therapieempfehlung aufscheinen, bringt eine Erwähnung in einer medizinischen Leitlinie stets einen Glaubwürdigkeitsbonus mit sich. Es soll daher der Frage nachgegangen werden, ob diese Arbeiten für sich eine Validität und Aussagekraft haben, die dies rechtfertigen könnte.

#### Wiesenauer et al. [27]

In einer **randomisierten Doppelblindstudie**

Randomisierte Doppelblindstudie

wurde bei 152 Patienten mit Sinusitis der therapeutische Effekt von unterschiedlichen homöopathischen Komplexmitteln im Vergleich zu Placebo untersucht. Die Autoren beschreiben zwar durchweg Verbesserungen, dokumentieren aber **keine signifikanten Unterschiede**

Keine signifikanten Unterschiede,

weder zwischen den 3 untersuchten Komplexmitteln noch im Vergleich zu Placebo. Dennoch gelangen die Autoren der Arbeit nicht dahin, den gebotenen Schluss zu ziehen, dass die Nullhypothese gescheitert ist, die Komplexmittel seien über Placebo hinaus wirksam.

#### Weiser et al. [28]

Diese randomisierte placebokontrollierte Doppelblindstudie sollte in einer **prospektiven Untersuchung**

Prospektive Untersuchung

die Überlegenheit eines homöopathischen Nasensprays bei chronischer Sinusitis zeigen. Die Ergebnisse der Gruppen lassen keine hinreichenden Unterschiede erkennen: Einerseits zeigten sich die deutlichsten Ergebnisse bei **subjektiven Bewertungen**

Subjektive Bewertungen

der Auswirkungen der Therapie auf Atembehinderung, Druckgefühl und Kopfschmerz, andererseits äußerten die Patienten der Placebogruppe die größere Zufriedenheit mit dem Therapieerfolg. Bei derartigen Outcomes, die möglicherweise lediglich Präferenzen der Probanden widerspiegeln, kann nicht auf einen therapeutischen Nutzen des Homöopathikums geschlossen werden.

#### Friese et al. [29]

Die Autoren geben an, dass ihnen der Wirksamkeitsnachweis für ein homöopathisches Kombinationspräparat bei akuter Rhinosinusitis gelungen sei. Doch auch diese doppelblinde placebokontrollierte Studie scheitert am wissenschaftlichen Einmaleins.

Das zunächst spektakulär erscheinende Ergebnis beruht nicht auf einer starken Wirksamkeit des untersuchten Komplexmittels, sondern ist ein statistisches Artefakt, das Ergebnis einer **hohen Ausfallrate**

Hohe Ausfallrate

in der Placebogruppe (88 %). Bei einer derart hohen Ausfallrate in einer der Gruppen – in der Homöopathiegruppe gab es nur einen einzigen Abbruch – fehlt einem Vergleich der Gruppenergebnisse jede reale Basis, er ist deshalb ohne jede Aussagekraft.

#### Grimaldi-Bensouda et al. [30]

Diese Arbeit entstammt dem von einem französischen Homöopathiehersteller finanzierten **EPI3-Projekt**

EPI3-Projekt,

einem Konglomerat verschiedener Verlaufs- bzw. Beobachtungsstudien. Sie betrachtet in einer Verlaufsstudie das Management von Infektionen der oberen Atemwege mit unterschiedlichen Therapieansätzen, u. a. der Homöopathie. Es handelt sich nicht um eine prospektiv-experimentelle Arbeit und ist damit systemisch zum Nachweis einer Evidenz für die Wirkung der Interventionen ohnehin nicht geeignet.

Methodisch fällt auf, dass die Beobachtungspunkte für den Verlauf einen, 3 und 12 Monate nach Therapiebeginn lagen, zu Zeiten also, an denen eine anfängliche Entzündung der oberen Atemwege völlig unabhängig von jeder Therapie i. d. R. ausgeheilt ist. Demgemäß sind hieraus nicht einmal Rückschlüsse auf Unterschiede verschiedener Therapieformen möglich [31].

#### Nayak et al. [32]

Hierbei handelt es sich um eine **prospektive Verlaufsstudie**

Prospektive Verlaufsstudie

ohne Vergleichsgruppe. Methodisch ist sie deshalb für den Schluss, dass die dargelegten positiven Effekte solche der homöopathischen Behandlung wären, nicht geeignet. Dass zudem die Diagnosestellung einer chronischen Sinusitis mittels konventioneller Röntgenaufnahme nach gültigen fachlichen Standards obsolet ist und dadurch nicht einmal ein eindeutiges Patientenkollektiv vorliegt, sei nur am Rande erwähnt.

##### Merke

Keine dieser in der S2k-Leitlinie erwähnten Studien zur Sinusitis legt nahe, dass die negative Gesamtevidenz zur Homöopathie einer Revision oder auch nur einer näheren Neubetrachtung bedürfte.

## Homöopathie in Leitlinien

Die zuvor genannten Beispiele zeigen, wie problematisch es ist, einzelne homöopathische Studien in Leitlinien aufzunehmen, ohne sie vorher kritisch auf Validität zu prüfen und vor dem Hintergrund der Gesamtevidenz unmissverständlich einzuordnen.

### Merke

- Die GRADE-Kriterien der Cochrane Collaboration gelten ausdrücklich für die medizinischen Empfehlungen der EbM – dies sind die Leitlinien –, nicht nur für die Qualitätsbewertung im Rahmen systematischer Übersichtsarbeiten.
- Leitlinien sind der „Outcome“ des Systems der EbM, das eigentliche Ziel des Konzepts: In ihnen wird die „jeweils verfügbare beste externe klinische Evidenz“ für die Behandlungspraxis zugänglich gemacht.
- Ein Dissens zwischen Gesamtevidenzen und Leitlinien dürfte sich demgemäß nicht ergeben.

Homöopathie dürfte die einzige Intervention sein, für die die EbM auf der höchsten Stufe der systematischen Reviews keine positive Gesamtevidenz belegt, die jedoch trotzdem ihren Niederschlag in EbM-Leitlinien findet. Unabhängig davon, ob solche Arbeiten direkt in Therapieempfehlungen einfließen oder lediglich Optionen sind, trägt jede nicht explizit bewertete Nennung der Homöopathie in Leitlinien der EbM zu einer **verzerrten Rezeption**

Verzerrte Rezeption

der Homöopathie in der Ärzteschaft bei. Die damit in Verbindung stehenden grundsätzlichen Fragen der Leitliniengewinnung sind einem anderen Kontext vorbehalten.

## Homöopathie und Antibiotika

Für den HNO-Bereich ist das Thema der Antibiotikaverordnung im Zusammenhang mit dem Resistenzproblem von hoher praktischer Bedeutung. Da die Homöopathie in letzter Zeit gezielt unter diesem Aspekt in den Fokus gerückt wird, sei dies näher betrachtet.

Dass die Vertreter der Homöopathie für ihre Methode unter der Flagge des **Antibiotikaresistenzproblems**

Antibiotikaresistenzproblem

werben, ist nicht neu [33]. Keimbedingte Erkrankungen spielen in den Prämissen der Homöopathie, namentlich im Konzept der „verstimmten geistigen Lebenskraft“ als ihrer einzigen ätiologischen Deutung eines Krankheitsgeschehens, keine Rolle. Die Mikrobiologie erfuhr erst nach Hahnemanns Tod durch die bahnbrechenden Arbeiten von Koch, Semmelweis und Pasteur eine wissenschaftliche Fundierung, was seinerseits als wesentliche Widerlegung des hahnemannschen Konstrukts gilt.

### Merke

- Es existieren keine belastbaren Ergebnisse aus randomisierten kontrollierten Studien dafür, dass Homöopathie eine Alternative zu Antibiotika sein könnte [34].
- Oft sind selbstlimitierende einfache Krankheitsbilder Untersuchungsgegenstand, bei denen ein Verlauf nicht auf eine spezifische Behandlung – gleich welcher Art – zurückgeführt werden kann.
- Auch werden gelegentlich Vergleiche zwischen Homöopathie und Antibiotika bei Indikationen angestellt, bei denen gar keine Antibiotika indiziert waren, also Placebo mit Placebo mit dem Ergebnis „vergleichbarer Unwirksamkeit“ getestet wurde.
- Es bleibt bei den Ergebnissen der systematischen Reviews, die kein Krankheitsbild – auch kein für Antibiotika indiziertes – identifiziert hatten, für die Homöopathie zur Behandlung geeignet gewesen wäre.

Es mag ein – bedenkliches – Zeichen für die ungebrochene **öffentliche Reputation**

Öffentliche Reputation

der Homöopathie trotz des Mangels an wissenschaftlichen Belegen sein, dass 2019 auch die politische Sphäre unhinterfragt dem Narrativ von „Homöopathie gegen Antibiotikaresistenzen“ gefolgt ist. Am 07.11.2019 hat der Bayerische Landtag auf Vorschlag des Gesundheitsausschusses beschlossen, in einer Studie die Möglichkeiten insbesondere des Einsatzes von Homöopathika zur Reduzierung der Antibiotikagaben untersuchen zu lassen.^11^

Diese Entscheidung ist nicht kompatibel mit der negativen Gesamtevidenz der Homöopathie. Borkens und Plasberg (2020) haben den Beschluss des Landtags unter dieser und auch unter ethischen Prämissen als verfehlt kritisiert [35].

## Informed Consent und Placeboproblematik

Nach wissenschaftlichem Erkenntnisstand ist die Homöopathie als Methode ohne spezifische, intrinsische Wirkung identifiziert. Das Geflecht der daraus folgenden patientenbezogenen, medizinethischen und **praktischen Implikationen**

Praktische Implikationen

ist dicht.

Homöopathie ist nach dem Selbstverständnis ihrer Proponenten keine Gesprächs- und ebenso wenig eine Placebotherapie, sondern eine spezifische Arzneimitteltherapie. Hahnemann selbst nahm dies ausdrücklich für seine Methode in Anspruch und baute dies auf seinem Konzept der Korrektur einer „verstimmten geistigen Lebenskraft“ durch eine „geistige Arzneikraft“ auf. Gerade dem spezifischen Arzneimittelkonzept widerspricht die negative Gesamtevidenz umfassend.

Insofern wäre es an sich müßig, auf die Problematik einer Verwendung von Homöopathie als Placebo in der ärztlichen Praxis näher einzugehen. Jedoch ist die Ansicht, dass Homöopathie als Placebomethode vertretbar sei, nicht gänzlich unplausibel, sodass die damit zusammenhängenden Fragen Beachtung erfordern.

Das Konzept des Informed Consent, des **informierten Einverständnisses**

Informiertes Einverständnis

über die Aspekte des Therapievorschlags, ist ein zentraler Gesichtspunkt neuzeitlicher Medizinethik und mit den Ethikrichtlinien des Weltärztebundes [36] auch verbindlich geworden. Die Ethikrichtlinien bezeichnen den Informed Consent zwischen Arzt und Patient über eine Therapieentscheidung, als „einen der zentralen Begriffe der heutigen ärztlichen Ethik“. Informed Consent bei einer Placebotherapie erfordert auf TherapeutInnen- wie auf PatientInnenseite ein wissendes Einverständnis darüber, dass es sich überhaupt um eine Placebotherapie handelt.

Ein Informed Consent in diesem Sinne macht eine solche Placebogabe nicht etwa inhaltsleer. Frühere wie neuere Untersuchungen, die die **medizinethische Problemlage**

Medizinethische Problemlage

dabei besonders betonen, bestätigen den psychotropen Effekt offener Placebos, also solcher, die dem Patienten gegenüber als solche deklariert wurden. Eine „gezielte Täuschung“, die stets unvertretbar ist, könnte damit hinfällig werden [37]. Auch im Falle der Homöopathie?

### Merke

- Die Homöopathie stellt hinsichtlich des Informed Consent eine besondere Problemlage dar.
- Sie ist mit so vielen Heils- und Heilungsversprechen und einem dementsprechenden positiven Image verbunden, dass es kaum denkbar scheint, hier mit ausreichender Sicherheit einen Informed Consent zwischen den Therapiepartnern herzustellen.
- Das Narrativ einer „sanften, nebenwirkungsfreien und wirksamen Therapie“ schwingt stets mit.
- Eine reale, bewusste Akzeptanz und Wahrnehmung der Homöopathie als reines Placebo kann damit i. d. R. nicht vorausgesetzt werden.
- Es bleibt im Fall der Homöopathie, der ihre eigene allgemeine Reputation hier Grenzen setzt, stets bei einer immanenten Täuschung, die der Therapeut nicht ausschließen kann.

Dies heißt jedoch nichts anderes, als dass der einzige akzeptable Zustand – der/die TherapeutIn und der/die PatientIn gehen gleichermaßen und in gegenseitiger Akzeptanz vom Placebocharakter der Homöopathie aus – praktisch nicht herstellbar ist. Die anderen 3 Konstellationen – beide Parteien gehen von spezifischer Wirksamkeit aus oder aber jeweils eine – führt zum Fehlen eines informierten Einverständnisses. Beabsichtigt der/die TherapeutIn eine Placebogabe und geht der/die PatientIn davon aus, ein wirksames Arzneimittel zu erhalten, liegt gar eine aktive Täuschung vor. Es liegt auf der Hand, dass deshalb auch Dinge wie Verlegenheits- oder Wunschverschreibungen von Homöopathika anstelle einer **PatientInnenaufklärung**

PatientInnenaufklärung

über die **spezifische Wirkungslosigkeit**

Spezifische Wirkungslosigkeit

der Methode bzw. Nichtnotwendigkeit einer medikamentösen Therapie ausgeschlossen sind.

Dem wäre noch hinzuzufügen, dass nach Ansicht führender Placeboforscher der Anteil des eigentlichen Placeboeffekts an den gesamten Kontexteffekten vermutlich massiv überschätzt wird [38] und seriöse Forschung diskreditiert werde, wenn der Effekt dazu dienen soll, Pseudomethoden zu rechtfertigen [39].

## Homöopathie als Teil des Versorgungssystems

Der Dissens zwischen der gegenwärtig privilegierten Rolle der Homöopathie im Arzneimittel- und im Sozialrecht und der medizinischen Invalidität der Methode hat Verwerfungen im Versorgungssystem zur Folge, die bis zur Abrechnung ärztlich-homöopathischer Leistungen und darüber hinaus reichen.

Wenn Homöopathie nicht ohnehin schon ausschließlich als Privatleistung erbracht wird, können ÄrztInnen die Möglichkeit nutzen, die Quartalsbudgets für GKV-PatientInnen über **Homöopathie-Selektivverträge**

Homöopathie-Selektivverträge

zu vervielfachen.^12^

Seit 2005 besteht die Option, als homöopathischer Vertragsarzt/-ärztin von den Homöopathie-Selektivverträgen vieler GKV-Kassen und Kassenärztlichen Vereinigungen mit der Marketinggesellschaft des Deutschen Zentralvereins homöopathischer Ärzte zu profitieren (§ 73 c SGB V). Seit 2012 haben zudem die GKV-Kassen die – weithin genutzte – Option, Homöopathika im Rahmen von **Satzungsleistungen**

Satzungsleistungen

zu erstatten (3. GKV-Versorgungsstrukturgesetz, § 11 Abs. 6 SGB V), was zweifellos zur Steigerung der Nachfrage an Homöopathie durch GKV-PatientInnen beigetragen hat. Die Honorarsätze sind im Vergleich zu den Quartalsbudgets überaus attraktiv, sodass es lohnend scheint, die begrenzte Praxiszeit eher für die homöopathieaffine als für die „normalen“ Patientenschaft aufzuwenden.

Es kann dahingestellt bleiben, ob die Honorierung von „mehr Zeit“ über die Honorarsätze der Selektivverträge als pragmatisches Äquivalent dafür angesehen wird, dass im derzeitigen Abrechnungssystem die **Honorierung des PatientInnengesprächs**

Honorierung des PatientInnengesprächs

längst marginalisiert wurde.^13^ Eine Lösung des Problems zu geringer und zu gering honorierter Zeiten für die „sprechende und zuhörende“ Medizin liegt hierin jedenfalls nicht. Die Praxiszeit insgesamt vermehrt sich dadurch schließlich nicht, ebenso wenig wie die Zahl der niedergelassenen ÄrztInnen. Im Gegenteil, richtig bedacht, geht die besondere Honorierung von Zeitaufwand für die homöopathieaffine Patientengruppe erheblich zulasten der anderen PatientInnen, die keine homöopathischen Leistungen in Anspruch nehmen (wollen), denn für die steht ja dann noch weniger Zeit zur Verfügung. Eine „Verbesserung der Versorgung“, was einmal die Begründung für die Einführung der Selektivverträge war, liegt hierin sicher nicht. Dessen sollte man sich bewusst sein. Der Gesetzgeber sollte nicht versuchen, Lücken und Unzulänglichkeiten im Versorgungssystem über Privilegien für wissenschaftlich nicht valide Methoden zu schließen.

### Merke

In der Einbindung der Homöopathie in die Strukturen der Gesundheitsversorgung liegen massive, nicht zielführende Fehlanreize, die sich nicht allein in der allgemeinen Kritik der öffentlichen Privilegierung einer Methode ohne wissenschaftlichen Wirkungsnachweis erschöpfen.

## Ausblick

Das Ziel der EbM ist, die jeweils beste verfügbare externe Evidenz für die medizinische Praxis verfügbar zu machen. Dies bedingt eine **ständige Neubewertung**

Ständige Neubewertung

bisher akzeptierter medizinischer Verfahren. Damit soll die Lücke zwischen wissenschaftlicher Erkenntnislage und medizinischer Praxis so klein wie möglich gehalten werden. Im Gegensatz dazu haben die Ergebnisse empirischer Forschung zur Homöopathie praktisch keine Konsequenzen für deren therapeutische Praxis und generell für ihren Anspruch, Teil der Medizin zu sein.

Obwohl die Proponenten der Homöopathie den Evidenzbegriff für sich zu reklamieren suchen, ist nicht erkennbar, dass sie die konsensuale Gesamtevidenz anerkennen oder irgendwelche praktischen Konsequenzen aus der Studienlage ziehen. Ein solcher Umgang mit der EbM im Sinne einer **praxisirrelevanten Bestätigungsforschung**

Praxisirrelevante Bestätigungsforschung

ist abzulehnen, da er das Instrument der klinischen randomisierten experimentellen Studie als einem der wichtigsten Messinstrumente der medizinischen Empirie entwertet.

Der Hals-Nasen-Ohren-ärztliche Fachbereich ist vielfach mit Krankheitsbildern konfrontiert, die einen selbstlimitierenden Verlauf haben – ein typischer Fallstrick für die Fehlannahme, hier habe eine vorher stattgefundene homöopathische Intervention „gewirkt“. Die Patientenschaft darf nicht in einer solchen Annahme belassen werden. Gesundheitskompetenz, die nach wie vor in erster Linie von ÄrztInnen in der täglichen Praxis vermittelt wird, entsteht nicht durch Fehlinformation, die gerade bei der so sehr mit **Falschannahmen**

Falschannahmen

besetzten Homöopathie auch in **Nichtinformation**

Nichtinformation

bestehen kann.

In der Nennung von indikationsbezogenen homöopathischen Studien in Leitlinien der evidenzbasierten Medizin sehen die Verfasser angesichts der negativen Gesamtevidenz einen gewissen Dissens, der aber in einem anderen Kontext zu diskutieren wäre. Problematisch erscheint, wenn im Outcome des EbM-Prozesses einzelnen Arbeiten wieder Relevanz zugesprochen wird, die zu einer Methode gehören, die vorher bei der Prüfung ihrer Gesamtevidenz den Validitätsmaßstäben der EbM nicht standgehalten hat.

Die medizinethischen Probleme einer homöopathischen Behandlung sind gravierend. Primär trifft den/die TherapeutIn die **Aufklärungspflicht**

Aufklärungspflicht

darüber, dass es sich um einen Therapieversuch mit einem Mittel ohne belegte Wirkung, also einem Placebo, handelt. Hiermit einen wirklichen Informed Consent zu erreichen, scheint aber im Fall der Homöopathie nahezu unmöglich. Die Homöopathie ist vielfältig mit Heils- und Wirkungsversprechen aller Art verbunden, die ihre allgemeine Wahrnehmung als wirksame medizinische Option prägen. Es wird in der Praxis deshalb fast nie gelingen, gegen diese allgemeine Wahrnehmung ein wirklich fundiertes informiertes Einverständnis zum Einsatz von Homöopathika als Placebo herbeizuführen. Eine homöopathische Behandlung, die sich – zumal entgegen dem Selbstverständnis der Methode als spezifische Arzneimitteltherapie – als Placebotherapie rechtfertigen will, ist damit in aller Regel nicht vertretbar und ist zudem auch nicht kompatibel mit dem eigenen Anspruch der Homöopathie, eine spezifische Arzneimitteltherapie zu sein.

Die gesetzliche Privilegierung der Homöopathie durch das Arzneimittelgesetz 1978, die der Methode Sonderrechte zur Erlangung der Arzneimitteleigenschaft einräumt, entbehrt längst jeglicher rationalen Grundlage, wie man sie vor 40 Jahren noch vermutet haben mag, und entfaltet nur noch formale Bedeutungskraft.

Ein Bekenntnis zu einer modernen **wissenschaftsbasierten Medizin**

Wissenschaftsbasierte Medizin,

die sich zeitgemäßer medizinischer Ethik sowie dem wissenschaftlich begründeten Erkenntnisgewinn auf der Basis der EbM verpflichtet fühlt, schließt nach derzeitigem Erkenntnisstand wissenschaftlich invalide, empirisch als unbelegt geltende Methoden wie die Homöopathie als therapeutische Optionen aus.

## Fazit für die Praxis

- Homöopathie ist keine Therapieoption für die medizinische Praxis.
- Das Bewusstsein dafür, dass für die Homöopathie ein umfassender Body of Evidence existiert, der über Jahrzehnte hinweg mittels etlicher systematischer Übersichtsarbeiten validiert worden ist, scheint angesichts der Faktenlage erstaunlich gering ausgeprägt.
- Es ist jedoch geltender wissenschaftlicher Konsens, dass keine belastbare Evidenz für die spezifische Wirksamkeit homöopathischer Behandlungen existiert.
- Die fehlende Gesamtevidenz für eine spezifische Wirkung homöopathischer Therapieansätze bei irgendeiner Indikation macht eine Berufung auf einzelne scheinbar bestätigende Arbeiten, geschweige denn individuelle Einzelerfahrungen, als Basis für eine Therapieentscheidung nach aktuellen medizinischen Maßstäben obsolet.

## CME-Fragebogen

Was versteht man unter dem Begriff „Body of Evidence“ für eine Intervention (Methode/Mittel)?

Die Gesamtevidenz für eine Intervention

Die Gesamtheit aller Erkenntnismaterialien zu einer Intervention

Die Zusammenfassung von Primäruntersuchungen zu Metadaten

Ein wissenschaftlich begründetes formales Konsensusverfahren

Ein systematisches, mehrstufiges Befragungsverfahren mit Rückkopplung

Welchen Zweck hat die Einordnung von Erkenntnismaterialien in Evidenzklassen?

Sie ermöglicht die Auswahl von Erkenntnismaterialen für einen Evidenzbeleg.

Sie lässt auf die Gesamtevidenz einer Intervention zurückschließen.

Sie definiert die positiven Belege aus dem Erkenntnismaterial für eine Intervention.

Sie definiert die negativen Belege aus dem Erkenntnismaterial für eine Intervention.

Sie ordnet wissenschaftliches Erkenntnismaterial nach methodischen Gesichtspunkten.

Wie definiert sich der Begriff einer Gesamtevidenz im Sinne der evidenzbasierten Medizin (EbM) für eine Intervention (Methode/Mittel)?

Als das Ergebnis systematischer Bewertung des Body of Evidence

Sie ist identisch mit dem Body of Evidence

Als die Meinung der führenden Experten eines Fachgebiets

Als das Ergebnis der zuletzt erschienenen Arbeiten eines Fachgebiets

Als das Ergebnis einer qualitativ guten randomisierten kontrollierten Studie für eine Intervention

Die interne Validität einer wissenschaftlichen Studie ist ein wesentliches Beurteilungskriterium bei der Evidenzfindung. Wie wird diese im Kontext medizinwissenschaftlicher Studien definiert?

Als das Maß, mit dem die Studie zum gewünschten Ergebnis führt

Als ihre statistische Belastbarkeit in Abhängigkeit von den Kohortengrößen

Als Grad der methodischen Zuverlässigkeit ihrer Konzeption, Durchführung und Auswertung

Als gegeben, wenn eine Kontrollgruppe mitgeführt worden ist

Als abhängig davon, ob Interessenkonflikte vollständig und richtig angegeben worden sind

Systematische Reviews gelten in der evidenzbasierten Medizin (EbM) als höchste Referenz für die Begründung von Evidenz einer Intervention (Methode/Mittel). Was ist ihr wesentliches Merkmal?

Sie erstellen eine summarische Übersicht über vorhandene Studien.

Sie prüfen die Schlussfolgerungen in randomisierten kontrollierten Studien auf Plausibilität.

Sie bewerten Arbeiten höherer Evidenzklassen qualitativ und leiten eine Gesamtevidenz ab.

Sie konzentrieren sich auf Erkenntnismaterialen mit für die jeweilige Intervention positiven Ergebnissen.

Sie sind mit Metaanalysen gleichzusetzen.

Der Risk of Bias ist eine definierte Größe im Prozess der Erstellung systematischer Reviews. Wie definiert er sich nach den Grundsätzen der evidenzbasierten Medizin (EbM)?

Er entspricht dem Risiko, aus dem Body of Evidence die falschen Materialien auszuwählen.

Er beurteilt sich nach dem Grad der internen Validität einer wissenschaftlichen Arbeit.

Er bewertet das Risiko von Voreingenommenheit bei den Verfassern einer wissenschaftlichen Arbeit.

Er spiegelt das Risiko einer Verzerrung der Studienlage durch Nichtveröffentlichung wichtiger Forschungsergebnisse.

Er misst den Grad der Übereinstimmung wissenschaftlicher Ergebnisse mit vorhandenen gesicherten Erkenntnissen (externe Validität).

Die Gesamtevidenz der Homöopathie ist umfassend validiert. Worauf stützt sich diese Aussage?

Auf die im Ergebnis einheitlichen Schlussfolgerungen systematischer Reviews der letzten Jahrzehnte.

Auf die Tatsache, dass weltweit Millionen Menschen die Homöopathie anwenden.

Auf die immer wieder präsentierten indikationsbezogenen Studien mit signifikanten Ergebnissen für die Homöopathie.

Auf den Umstand, dass Homöopathie bis heute existent und beliebt ist.

Auf die Erfahrungstatbestände zahlreicher homöopathischer Therapeuten.

Leitlinien der Arbeitsgemeinschaft der Wissenschaftlichen Medizinischen Fachgesellschaften e.V. (AWMF) und von Fachgesellschaften gewinnen zunehmende Bedeutung für die ärztliche Praxis. Welche Rolle kommt ihnen dabei zu?

Sie stellen „medizinische Leitlinien“ im Sinne der evidenzbasierten Medizin (EbM) dar und vermitteln die jeweils beste verfügbare externe Evidenz für Therapieentscheidungen.

Sie sind die Zusammenfassung des Body of Evidence für medizinische Interventionen.

Sie repräsentieren Praxiserfahrungen der an der Erarbeitung der Leitlinien beteiligten Experten.

Sie stellen Pflichtvorgaben für die ärztliche Praxis dar.

Sie stehen in keinem Zusammenhang mit dem Prozess der EbM und sind freie Empfehlungen führender Experten.

Ihnen wird eine neue einzelne Studie zur Homöopathie mit „signifikantem“ Ergebnis zugunsten der Nullhypothese (Überlegenheit gegen Placebo) vorgelegt. Welche erste Einschätzung würden Sie verwerfen?

Im Hinblick auf die gesicherte Gesamtevidenz ist es nicht gerechtfertigt, eine Therapierelevanz anzunehmen.

Es handelt sich mit hoher Wahrscheinlichkeit um eine falsch-positive Studie (Alpha-Fehler).

Signifikanz bedeutet nicht, dass ein klinischer Effekt von einer für die Patienten relevanten Stärke vorliegt.

Für die vorgestellte Indikation ist eine Evidenz für einen homöopathischen Therapieansatz damit belegt.

Es handelt sich mit hoher Wahrscheinlichkeit um eine Signifikanz, die auf methodische/statistische Mängel zurückzuführen ist.

Sie erwägen eine Verordnung von Homöopathika als Placebo bei einer selbstlimitierenden leichten Erkrankung, weil Ihr Patient/Ihre Patientin eine Medikation wünscht. Wie entscheiden Sie unter medizinethischen Aspekten?

Das Homöopathikum kann verordnet werden, weil kein Schadenspotenzial erkennbar ist.

Das Homöopathikum kann zur Wahrung des Vertrauensverhältnisses zum Patienten/zur Patientin verordnet werden.

Das Homöopathikum kann verordnet werden, weil ohnehin jeder um die medizinische Irrelevanz der Homöopathie weiß.

Ohne überzeugenden Informed Consent über die medizinische Irrelevanz der Homöopathie ist die Verschreibung i. d. R. zu versagen.

Das Homöopathikum kann verordnet werden, damit die Erstattung durch die gesetzliche Krankenversicherung (GKV) erfolgen kann.

## Footnotes

1 Im Sinne einer konsequentialistischen Handlungsethik, die auf eine Vergrößerung des allgemeinen Wohls (durch sinnvolle und zweckrationale medizinische Interventionen) abzielt.

2 Ähnlichkeits- und Potenzierungsprinzip werden hier als „Mindestkonsens“ für den Homöopathiebegriff unterstellt. „Die“ Homöopathie als einheitliche Methode existiert nämlich nicht, sondern ist längst in eine Vielzahl unterschiedlicher, teils sich elementar widersprechender Richtungen zerfallen. Es dürfte aber im gegebenen Kontext legitim sein, Ähnlichkeits- und Potenzierungsprinzip bei einer Betrachtung der Homöopathie per se als kleinste gemeinsame Nenner anzusehen.

3 Die „zweite Chance“ ist für die Homöopathie umso vorteilhafter, als dass sie „Vorhandenes“ untersucht und – anders als bei der Neuentwicklung eines Mittels in der Pharmazie – nicht gehalten ist, vor jeglicher klinischen Prüfung Pharmakinetik und Pharmakodynamik ihrer Mittel schlüssig zu beschreiben. Dazu wäre sie nicht imstande.

4 Entsprechend der Maxime von Ray *Hyman*: „Don’t try to explain HOW something works until you find out THAT it works.“

5 *Metaanalysen* fassen Datenbestände aus Studien für eine summarische methodisch-statistische Auswertung zusammen. Dies ist wegen der Heterogenität der Daten und Methoden jedoch oft nicht möglich, so dass in *systematischen Reviews* angestrebt wird, über die inhaltlich-qualitative Bewertung der Erkenntnismaterialen zu einer Gesamtbeurteilung auf der Metaebene zu gelangen. Auch die zusammenfassende Betrachtung mehrerer Reviews ist dabei ein üblicher Vorgang.

6 *Mathie* (2014): „Keine (!) Studie wurde mit ‚A‘ bewertet (geringes Risiko eines Bias insgesamt), d. h. keine erfüllte die Kriterien für alle sieben Bewertungsbereiche.“ *Mathie* (2018): „Künftige ‚*other-than-placebo‘*-kontrollierte Studien in der Homöopathie sollten so weit wie möglich darauf abzielen, sowohl die interne Validität als auch die externe Validität zu fördern.“.

7 Vgl. hierzu den persönlichen Bericht von Prof. Paul Glasziou, dem Arbeitsgruppenleiter für die Bewertung des NHMRC-Reviews, über seine Arbeit in diesem Zusammenhang: https://blogs.bmj.com/bmj/2016/02/16/paul-glasziou-still-no-evidence-for-homeopathy/.

8 House of Commons, Science and Technology Committee (2009), Russische Akademie der Wissenschaften (2017), European Academies Science Advisory Council EASAC (2017), National Health Services of the UK (2018) u. a.

9 Ioannidis: „*Claimed research findings may often be simply accurate measures of the prevailing bias*“ [19]. Ebenso mittels einer neueren Methode der p‑Kurven-Analyse zur Unterscheidung von statistischen Artefakten von realen Effekten: Reisman et al. [20]; Prochaska [21] (Masterarbeit an der Universität Wien)

10 Vgl. Novella (2016): „*Even if you take a strict EBM approach and do not consider scientific plausibility, the evidence does not support the conclusion that homeopathy works for anything. There is not one indication for which any homeopathic treatment has been reliably shown to work, if you use a fair and reasonable threshold of evidence.*“ [22]

11 „Die Staatsregierung wird aufgefordert, durch eine Studie zu untersuchen bzw. untersuchen zu lassen, wie ein reduzierter Antibiotikaeinsatz im medizinischen Bereich realisiert werden kann. Dabei soll auch und insbesondere die Rolle alternativmedizinischer Methoden in den Blick genommen werden. Auch soll in diesem Zusammenhang eine mögliche positive Rolle von ggf. ergänzend verabreichten homöopathischen Präparaten beleuchtet werden.“ (http://www1.bayern.landtag.de/www/ElanTextAblage_WP18/Drucksachen/Basisdrucksachen/0000002000/0000002418.pdf.)

12 Einstündige Erstanamnese/Patientengespräch: 90 €. Repertorisation (Mittelfindung) und Analyse:45 €. Zwei Folgeanamnesen von je einer halben Stunde: 45 €. Beratungen von je 10 min zusätzlich je 10 € (Stand 2018).

13 Wobei allerdings auch nicht aus dem Auge verloren werden sollte, dass seinerzeit bei der Reform der Abrechnungsmodalitäten die „Gesprächsziffern“ zwar entfallen waren, aber kalkulatorisch in die Sachziffern eingerechnet wurden, was u. a. ein Wunsch der Ärzteschaft war, um zu einfacheren Abrechnungsmodalitäten zu kommen. Dass sich dies sowohl in der Wahrnehmung („es gibt keine Gesprächsziffern mehr“) als auch durch die Praxis der Budgetierung stark relativiert hat, ist offensichtlich.
