# Erratum zu: Homöopathie – eine Therapieoption für die Praxis? Bewertung unter dem Blickwinkel der evidenzbasierten Medizin

**DOI:** 10.1007/s00106-021-01089-y

**Published:** 2021-07-19

**Authors:** Christian W. Lübbers, Udo Endruscheit

**Affiliations:** 1 HNO Weilheim, Pöltnerstraße 22, 82362 Weilheim, Deutschland; 2 Krayer Straße 227, 45307 Essen, Deutschland

**Erratum zu:**

**HNO 2021**

10.1007/s00106-021-01061-w

Der Artikel „Homöopathie – eine Therapieoption für die Praxis?“ von Christian W. Lübbers und Udo Endruscheit wurde ursprünglich am 19. Mai 2021 ohne „Open Access“ online auf der Internetplattform des Verlags publiziert. Die Autoren haben sich jedoch nachträglich für eine „Open Access“-Veröffentlichung entschieden. Das Urheberrecht des Artikels wurde deshalb am 16. Juni 2021 in © Der/die Autoren 2021 geändert.

Der Artikel wird nun unter der Creative Commons Namensnennung 4.0 International Lizenz veröffentlicht, welche die Nutzung, Vervielfältigung, Bearbeitung, Verbreitung und Wiedergabe in jeglichem Medium und Format erlaubt, sofern Sie den/die ursprünglichen Autor(en) und die Quelle ordnungsgemäß nennen, einen Link zur Creative Commons Lizenz beifügen und angeben, ob Änderungen vorgenommen wurden. Die in diesem Artikel enthaltenen Bilder und sonstiges Drittmaterial unterliegen ebenfalls der genannten Creative Commons Lizenz, sofern sich aus der Abbildungslegende nichts anderes ergibt. Sofern das betreffende Material nicht unter der genannten Creative Commons Lizenz steht und die betreffende Handlung nicht nach gesetzlichen Vorschriften erlaubt ist, ist für die oben aufgeführten Weiterverwendungen des Materials die Einwilligung des jeweiligen Rechteinhabers einzuholen. Weitere Details zur Lizenz entnehmen Sie bitte der Lizenzinformation auf http://creativecommons.org/licenses/by/4.0/deed.de.

